# Prevalence of Ineffective Haplotypes at the Rice Blast Resistance (*R*) Gene Loci in Chinese Elite Hybrid Rice Varieties Revealed by Sequence-Based Molecular Diagnosis

**DOI:** 10.1186/s12284-020-0367-x

**Published:** 2020-01-30

**Authors:** Gui Xiao, Jianyuan Yang, Xiaoyuan Zhu, Jun Wu, Bo Zhou

**Affiliations:** 1 State Key Laboratory of Hybrid Rice, Hunan Hybrid Rice Research Center, Changsha, Hunan China; 2 International Rice Research Institute, DAPO Box 7777, Metro Manila, Philippines; 3 Plant Protection Research Institute, Guangdong Academy of Agricultural Sciences, Guangzhou, 510640 Guangdong China

**Keywords:** Rice blast, Sequence-based diagnosis, Resistance gene, Avirulence gene, Rice breeding

## Abstract

Multiple haplotypes at the same rice blast *R*-gene locus share extremely high sequence similarity, which makes the gene diagnostic method using molecular markers less effective in differentiation from one another. The composition and distribution pattern of deployed *R* genes/haplotypes in elite rice varieties has not been extensively analyzed. In this study, we employed PCR amplification and sequencing approach for the diagnosis of *R*-gene haplotypes in 54 Chinese elite rice varieties. A varied number of functional and nonfunctional haplotypes of 4 target major *R*-gene loci, i.e., *Pi2/9*, *Pi5*, *Pik*, and *Pib*, were deduced by referring to the reference sequences of known *R* genes. Functional haplotypes accounted for relatively low frequencies for the *Pi2/9* (15%) and *Pik* (9%) loci but for relatively high frequencies for the *Pi5* (50%) and *Pib* (54%) loci. Intriguingly, significant frequencies of 33%, 39%, 46% of non-functional haplotypes at the *Pi2/9*, *Pik*, and *Pib* loci, respectively, with traceable original donors were identified, suggesting that they were most likely unintentionally spread by using undesirable donors in various breeding programs. In the case of *Pi5* locus, only a single haplotype, i.e., *Pi5* was identified. The reactions of 54 rice varieties to the differential isolates were evaluated, which showed a good correlation to the frequency of cognate avirulence (*Avr*) genes or haplotypes in the differential isolates. Four *R* genes, i.e., *Pi2*, *Piz-t*, *Pi50*, and *Pikm* were found to contribute significantly to the resistance of the elite rice varieties. Other two genes, *Pi9* and *Pikh*, which were not utilized in rice varieties, showed promising values in breeding durable resistance due to their high resistance frequencies to the contemporary rice blast population. The sequence-based molecular diagnosis provided a promising approach for the identification and verification of haplotypes in different *R*-gene loci and effective *R* genes valuable for breeding durable rice resistance to rice blast.

## Background

Rice is a staple diet for nearly half of the global population (Harlan 1998; Skamnioti and Gurr 2009), and its increase in production to meet the ever-increasing population faces many challenges. Breeding high yield rice varieties, especially hybrid rice is one of the promising methods which has made a great contribution to safeguarding the food supply around the world (Tester and Langridge 2010). However, the high-yield hybrid rice is now seriously threatened by specific biotic stresses (blast, brown planthopper, bacterial blight, false smut, and so on) (Khush and Jena 2009; Wang and Valent 2017). Apart from focusing on yield potential, it is essentially important in improving the adaptability, resistance to biotic and abiotic stresses of rice.

Rice blast, caused by the fungal pathogen *Magnaporthe oryzae* (*M. oryzae*), is the most devastating rice disease and conspicuously reduces rice yield and grain quality (Kush and Jena 2009). The rice and rice blast system belong to a typical gene-for-gene system (Flor 1971), in which the host resistance (*R*) genes show functional correspondence to their cognate pathogen avirulence (*Avr*) genes (Orbach et al. 2000; Valent and Khang 2010). The co-evolution and interaction of *R* and *Avr* gene raises the possibility of a gene-specific arms race leading to diversification of both *R* and *Avr* genes (Dodds et al. 2006). Nine rice blast *Avr* genes have been cloned to date (Wang et al. 2017). The direct and indirect interaction models between *R* and *Avr* proteins were illustrated (Li et al. 2009; Yoshida et al. 2009; Kanzaki et al. 2012; Wu et al. 2015; Zhang et al. 2015; Ray et al. 2016). Field efficacy of any *R* gene in rice varieties is proposed to depend on the frequency of its cognate *Avr* gene in the rice blast pathogen population, which provides a basis of *Avr*-gene based diagnosis for the deduction of effectiveness of *R* genes (Selisana et al. 2017; Olukayode et al. 2019).

On the other hand, over 105 major rice blast *R* genes have been identified and 32 of them have been molecularly characterized (Li et al. 2017; Wang et al. 2017; Zhao et al. 2018). Many *R* genes are clustered at the same locus and allelic to each other. For instance, *Pi2*, *Piz-t*, *Pi9* and *Pi50* are allelic at the *Pi2/9* locus (Qu et al. 2006; Zhou et al. 2006; Su et al. 2015). *Pik*, *Pikp*, *Pikm*, *Pikh*, *Pi1* and *Pi7* are allelic at the *Pik* locus (Ashikawa et al. 2008; Yuan et al. 2011; Zhai et al. 2011; Hua et al. 2012). *Pish*, *Pi35* and *Pi37* are allelic at *Pish* locus (Lin et al. 2007; Takahashi et al. 2010; Fukuoka et al. 2014). Moreover, a limited number of sequence differences between these *R*-gene alleles were found to determine their specificities against distinct sets of rice blast isolates (Zhou et al. 2006; Fukuoka et al. 2014; Su et al. 2015). It was also found that functional and non-functional *R*-gene haplotypes at the same locus from resistant and susceptible rice varieties were distinguished by a few sequence changes (Bryan et al. 2000; Su et al. 2015). The fact that non-functional and functional *R*-gene haplotypes with distinct resistance specificities embedded in the same locus raises a reasonable concern about the sensitivity and reliability of the use of *R*-gene specific or linked markers in the diagnosis of the respective *R* genes present in novel germplasm. In this study, we employ a method combining PCR amplification and subsequent amplicon sequencing of portions of known rice blast *R* genes determining their function and resistance specificities for the diagnosis of *R*-gene haplotypes present in elite rice varieties. The function and the resistance specificities of *R*-gene haplotypes in these varieties are to be further validated using differential rice blast isolates with the reference of the presence of *Avr* genes.

## Methods

### Plant Materials

A total of 54 rice accessions were collected in this study. Forty of them were requested from the rice germplasm bank of Hunan Academy of Agricultural Sciences and the other 14 accessions were bought from the seed market. The detail information including name, promotion area and year, female and male parents for all these 54 rice accessions were listed in Additional file 1: Table S1. Eleven IRBLs, IRBLb-B, IRBLks-F5, IRBLkp-K60, IRBLkh-K3, IRBLi-F5, IRBL5-M, IRBLz5-CA, IRBLzt-T, IRBL9-W, IRBLz-Fu and IRBL1-CL, and the rice variety CO39 and NIL-e1 (monogenic line of *Pi50*) (Su et al. 2015) were also used in this study. Plants were grown in greenhouses at Hunan Hybrid Rice Research Institute.

### Fungal Materials and Disease Evaluation

Twenty seven *M. oryzae* isolates were used for disease evaluation and *Avr* gene diagnosis in this study. 26 of them were collected from Guangdong province which can represent the field population of *M. oryzae* in South China and one was collected from Hubei province, China. The name, collection year and place of these isolates were listed in Additional file 2: Table S2.

Rice seedlings at the 3–4 leaf stage were inoculated using these 27 isolates. The spore suspension with 10^5^ spores/ml was applied to plants using an airbrush connected to a source of compressed air. After inoculation, plants were held in the dark room for 24 h with 95–100% relative humidity and 24 °C. Then, plants were transferred to a greenhouse where the temperature was maintained at 25–28 °C and humidity was keep at 70–90%. Seven days after inoculation, disease symptoms were evaluated using a standard 0–9 scale (IRRI 2002), where 0–1 = highly resistant (HR), 2–3 = resistant (R), 4 = moderately resistant (MR), 5–6 = moderately susceptible (MS), 7 = susceptible (S), and 8–9 = highly susceptible (HS).

### Molecular Characterization of Haplotypes of Four *R* and Five *Avr* Genes/Locus

Genomic DNAs of both rice varieties and rice blast isolates were extracted using CTAB method and diluted to working concentration for further analysis. For the diagnosis of rice *R* genes, primers targeting the critical sequence portion capable for differentiating different haplotypes of *R*-gene loci were designed and optimized. For *Avr* genes in rice blast, primers targeting the entire genic sequence of each *Avr* gene were designed and optimized. The amplicons of both *R*-gene haplotypes and *Avr* genes were completely sequenced. The names, sequences, position and purpose of all the primers were shown in Additional file 3: Table S3. Polymerase chain reaction (PCR) was performed following the manufacturer’s instructions of the Q5 DNA polymerase (New England Biolabs).

### Sequence Data Analysis

The PCR amplification products were send to TSINGKE company (Changsha, China) for sequencing. Multiple alignment of DNA sequences was performed using the software SEQUENCHER (Gene Codes Corporation). The sequences of individual genes were downloaded from National Center for Biotechnology Information (NCBI) and used as reference sequence for alignment.

## Results

### Establishment of the method for the diagnosis of haplotypes at four rice blast *R*-gene loci

For the diagnosis of *R-*gene haplotypes, portions of known *R* genes that are capable for differentiating different haplotypes were targeted for PCR amplification and sequence analysis. Four *R*-gene loci, i.e., *Pi2/9*, *Pi5*, *Pik*, and *Pib*, representing those containing multiple functional and nonfunctional haplotypes in different rice varieties, were selected in this study. For the *Pi2/9* locus, an approximately 3.8-kb fragment corresponding to 3′ portion of *Nbs4-Pi2* at the *Pi2* locus (Genbank accession no.: DQ352453) flanked by Pi2/9-F3/R4 was targeted (Additional file 3: Table S3). It is worth noting that a prescreen step by PCR amplification was conducted using Pi2/9-DF1/DR1 to exclude those lines without positive PCR amplification as described previously (Xiao et al. 2017). For the *Pik* locus, the region coding for the HMA domain of *Pikp-1* (Genbank accession no.: HM035360) flanked by RGA4-F3/R3 was targeted (Additional file 3: Table S3). For the *Pi5* locus, a 740-bp fragment corresponding to the 3′ portion of *Pi5–2* (Genbank accession no.: EU869186) flanked by 09RL09-F2/R2 was targeted (Additional file 3: Table S3). For the *Pib* locus, the fragment corresponding to the 3′ portion of *Pib* (Genbank accession no.: AB013448) flanked by Pib-F4/R7 was targeted (Additional file 3: Table S3).

To verify the efficiency and specificity of PCR amplification of the target regions of different *R* gene, the Lijiangxintuanheigu (LTH)-derived International Rice Research Institute (IRRI) bred blast resistant lines (IRBLs) containing respective *R* genes were used for the validation and the derived amplicons were proceeded for sequencing confirmation. As Additional file 4: Figure S1 illustrated, the PCR amplification was strong and specific for those IRBLs. On the contrary, no PCR amplicons were obtained in CO39. These PCR-based markers were also found to effectively differentiate DNA samples from different varieties (Additional file 4: Figure S1). Sequences of the amplicons from IRBLs were confirmed to be identical to the respective known *R* genes, suggesting that these primers are working for the specific amplification of target portions of known *R* genes at different loci.

### Composition and Distribution of Different R-Gene Haplotypes in Elite Rice Varieties

A set of 14 rice varieties registered as top-50 varieties with large planting areas in 1994 and 40 hybrid rice varieties registered as top-50 hybrid rice varieties with large planting areas in 2015 were selected as representatives of elite rice varieties for the survey of the *R* genes in this study (Additional file 1: Table S1).

PCR-based analyses revealed that 24, 24, 27, and 42 varieties were resolved with expected amplicons for the *Pik*, *Pi2/9*, *Pi5* and *Pib* loci, respectively (Table 1). Amplicon sequencing and further cluster analyses revealed that these amplicons can be grouped into 5, 4, 1, and 2 haplotypes for the *Pik*, *Pi2/9*, *Pi5*, and *Pib* loci, respectively, indicating that many varieties most likely each contain the same *R*-gene haplotype at these 4 loci. The composition of the *R*-gene haplotypes at 4 loci was further identified by sequence comparison as follows:

**Table 1:** The PCR and sequencing results of different haplotypes of 4 *R*-gene loci in 54 rice varieties. PCR: Polymerase chain reaction; +: PCR positive; −: PCR negative; LTH: Lijiangxintuanheigu

Varieties	PCR results				Sequencing results			
	*Pik*	*Pi5*	*Pi2/9*	*Pib*	*Pik*	*Pi5*	*Pi2/9*	*Pib*
Shanyou 63	–	+	–	+	–	*Pi5–2*	–	*Pib-GII*
Zhefu 802	+	–	+	–	*Pikm1-LTH*	–	*Pi2/9-V20*	–
Zhe 773	+	–	+	+	*Pikm1-LTH*	–	*Pi2/9-V20*	*Pib-GII*
Wuyugeng No.3	–	–	–	–	–	–	–	–
Shanyou 46	–	–	–	+	–	–	–	Heterozygote (*Pib*/*Pib-GII*)
Gengxian 89	–	–	–	+	–	–	–	*Pib*
Weiyou 46	+	–	+	+	*Pikm1-LTH*	–	*Pi2/9-V20*	Heterozygote (*Pib*/*Pib-GII*)
Weiyou 77	+	+	+	+	*Pikm1-LTH*	*Pi5–2*	*Pi2/9-V20*	Heterozygote (*Pib*/*Pib-GII*)
Wuyugeng No.2	+	+	–	–	*Pik-1*	*Pi5–2*	–	–
Qishanzhan	–	+	–	+	–	*Pi5–2*	–	*Pib*
Xiangzaoxian No.7	+	–	–	+	*Pikm1-LTH*	–	–	*Pib-GII*
Ewan No.5	–	–	–	–	–	–	–	–
Eyi 105	–	–	–	+	–	–	–	*Pib-GII*
Xiushui 122	–	–	–	–	–	–	–	–
Shenliangyou 5814	–	–	–	+	–	–	–	*Pib-GII*
Y liangyou No.1	–	–	–	+	–	–	–	*Pib-GII*
Wuyou 308	–	+	+	+	–	*Pi5–2*	*Pi2/9-V20*	*Pib*
Tianyouhuazhan	+	+	+	+	*Pi7–1*	*Pi5–2*	*Pi2*	*Pib*
Yangliangyou No.6	+	+	–	+	*Pikm1-LTH*	*Pi5–2*	–	Heterozygote (*Pib*/*Pib-GII*)
Chuanyou 6203	+	+	–	+	*Pikm1-TS*	*Pi5–2*	–	*Pib*
Gangyou 188	+	+	–	+	*Pik-1-Shin2*	*Pi5–2*	–	Heterozygote (*Pib*/*Pib-GII*)
Wuyouhuazhan	–	+	+	+	–	*Pi5–2*	*Pi2*	*Pib*
C liangyouhuazhan	+	+	+	+	*Pikm1-LTH*	*Pi5–2*	Heterozygote (*Pi2/9-V20/Pi2*)	*Pib*
Xinliangyou No.6	–	+	–	+	–	*Pi5–2*	–	Heterozygote (*Pib*/*Pib-GII*)
Y liangyou 5867	–	–	–	+	–	–	–	*Pib-GII*
Zhongzheyou No.8	–	+	–	–	–	*Pi5–2*	–	–
Zhongzheyou No.1	–	+	–	+	–	*Pi5–2*	–	*Pib*
Xinrongyouhuazhan	–	+	+	+	–	*Pi5–2*	*Pi2*	*Pib*
Yueyou 9113	+	–	–	+	*Pikm1-LTH*	–	–	*Pib*
Rongyou 225	–	+	+	+	–	*Pi5–2*	*Pi2/9-V20*	Heterozygote (*Pib*/*Pib-GII*)
Rongyouhuazhan	–	+	+	+	–	*Pi5–2*	Heterozygote (*Pi2/9-V20/Pi2*)	Heterozygote (*Pib*/*Pib-GII*)
Liangyou 6326	–	+	–	+	–	*Pi5–2*	–	Heterozygote (*Pib*/*Pib-GII*)
H you 518	–	–	–	+	–	–	–	*Pib*
Luliangyou 996	+	–	+	+	*Pikm1-LTH*	–	*Pi2/9-V20*	*Pib-GII*
Guangliangyouxiang 66	–	–	–	+	–	–	–	Heterozygote (*Pib*/*Pib-GII*)
C liangyou 396	+	+	+		*Pikm1-LTH*	*Pi5–2*	*Pi2/9-V20*	–
Wandao153	+	–	–	+	*Pikm1-LTH*	–	–	*Pib*
Fengliangyou No.4	–	+	–	+	–	*Pi5–2*	–	Heterozygote (*Pib*/*Pib-GII*)
F you 498	+	+	+	+	*Pikm1-LTH*	*Pi5–2*	*Piz-t*	*Pib-GII*
C liangyou 343	+	–	+	+	*Pikm1-LTH*	–	*Pi2/9-V20*	*Pib*
Yueyou518	–	–	–	+	–	–	–	*Pib*
Wufengyou T025	+	–	+	–	*Pikm1-LTH*	–	*Pi2/9-V20*	–
Taiyou 390	+	–	+	+	Heterozygote (*Pi7–1*/*Pikm1-LTH*)	–	*Pi2/9-V20*	*Pib*
Tanliangyou83	+	+	+	+	*Pikm1-LTH*	*Pi5–2*	*Pi50*	*Pib-GII*
Tianfengyou 316	+	–	+	+	Heterozygote (*Pi7–1*/*Pikm1-LTH*)	–	*Pi2/9-V20*	*Pib*
Y liangyou 9918	–	+	–	–	–	*Pi5–2*	–	–
C liangyou 608	+	–	+	–	*Pikm1-LTH*	–	*Pi2/9-V20*	–
Liangyou 688	–	+	+	–	–	*Pi5–2*	*Pi2*	–
Huiliangyou 996	+	+	–	+	*Pikm1-LTH*	*Pi5–2*	–	*Pib*
Tianyou 998	–	–	+	–	–	–	*Pi2/9-V20*	–
Zhuliangyou 819	–	–	+	+	–	–	*Pi2/9-V20*	*Pib-GII*
Y liangyou No.6	–	+	–	+	–	*Pi5–2*	–	*Pib-GII*
Fengyuanyou 299	+	–	+	+	*Pikm1-LTH*	–	*Pi2/9-V20*	Heterozygote (*Pib*/*Pib-GII*)
Dexiang 4103	+	+	–	+	–	*Pi5–2*	–	*Pib-GII*

**Fig. 1:**
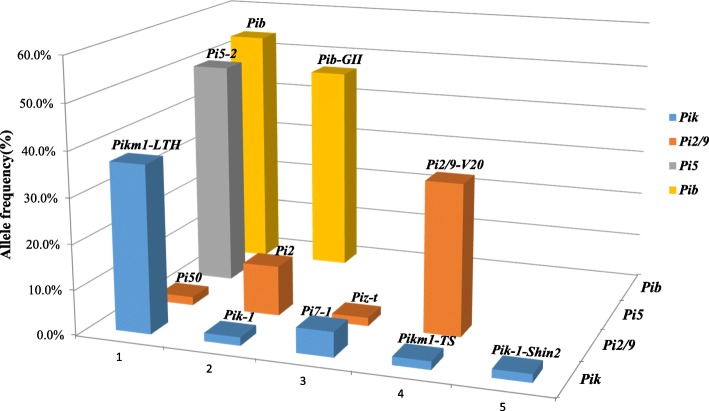
Frequency of different haplotype in 4 *R*-gene loci. The frequency of alleles was calculated based on percentage of the number of varieties containing the respective *R*-gene haplotypes in 54 rice varieties. The designation of *R*-gene haplotypes on the top of each column can be found in Table 1. Different *R*-gene loci are indicated in different colored boxes

The *Pik* locus: 5 haplotypes corresponded to *Pikm1-LTH* (Genbank accession no.: ANO81532), *Pik-1* (Genbank accession no.: HM048900), *Pi7–1* (Genbank accession no.: HQ660231), *Pikm1TS* (Genbank accession no.: AB462256), and *Pik-1-Shin2* (Genbank accession no.: ADE80950). Out of these 5 *Pik* haplotypes, *Pikm1-LTH* and *Pik-1-Shin2*, are most likely non-functional since their respective varieties LTH and Shin2 were characterized to be susceptible. Surprisingly, *Pikm1-LTH* was found disproportionally in 20 varieties representative of a frequency of 37% in varieties (Fig. 1 and Table 1). By including Gangyou188 containing *Pik-1-Shin2* and another 30 varieties without any *Pik* haplotypes (Fig. 1 and Table 1), approximately 94% of 54 tested varieties either contain non-functional *Pik* haplotypes or do not contain any *Pik* haplotypes. On the contrary, *Pik-1*, *Pikm1-TS*, and *Pi7–1* were respectively found in 1, 1, and 3 varieties including 2 heterozygous with *Pikm1-LTH*, representing extremely low frequencies in the elite rice varieties (Table 1 and Additional file 5: Figure S2).

The *Pi2/9* locus: 4 haplotypes corresponded *Pi2/9-V20*, a novel *Pi2/9* haplotype (Genbank accession no.: MN630588), *Pi2* (Genbank accession no.: DQ352453), *Pi50* (Genbank accession no.: KP985761), and *Piz-t* (Genbank accession no.: DQ352040). *Pi2/9-V20* was identified in 18 varieties representative of an approximately 33% of frequency in varieties (Table 1 and Fig. 1). It was further found that *Pi2/9-V20* was most likely non-functional since no strong association between its presence and the resistance of the respective variety against differential isolates was identified (Table 1 and Additional file 6: Table S4). Taken together, almost 89% of 54 varieties either contain non-functional *Pi2/9* haplotype or do not contain *Pi2/9* haplotypes. On the contrary, *Pi50*, *Piz-t*, and *Pi2* were respectively identified in 1, 1, and 6 varieties including 2 heterozygous with *Pi2/9-V20*, representing very low frequencies in the elite varieties (Table 1 and Additional file 5: Figure S2).

The *Pi5* locus: the single haplotype corresponded to *Pi5–2* (Genbank accession no.: EU869186), which was identified in 27 rice varieties representative of a frequency of 50% in elite varieties (Table 1 and Fig. 1).

The *Pib* locus: 2 haplotypes corresponded to *Pib-GII* (Genbank accession no.: MN630588) and *Pib* (Genbank accession no.: AB013448). *Pib* was identified in 29 varieties representative of an approximately 53.7% of frequency (Table 1 and Fig. 1). *Pib-GII*, a non-functional haplotype as described previously (Olukayode et al. 2019), was identified in 25 rice varieties (Table 1). It is worth noting that 12 varieties were found to be heterozygous with both *Pib* and *Pib-GII* (Table 1).

### The Composition and Distribution of Haplotypes of Different *Avr* Genes in Differential Isolates Used for the Resistance Evaluation of Elite Rice Varieties

A set of 27 *M. oryzae* isolates likely representative of the contemporary rice blast population was used as differential isolates for the resistance evaluation of 54 rice varieties (Additional file 2: Table S2). It was illustrated that the *Avr* gene or its haplotype could be used to diagnose the avirulence of the respective isolate to the rice variety containing the cognate *R* gene (Selisana et al. 2017). We conducted the analysis of the composition and distribution of 5 *Avr* genes and their haplotypes in these 27 differential isolates detailed as follows:

*AvrPii*: it was found only in the isolate 98–288 based on the PCR amplification (Table 2). Amplicon sequencing further verified that it was identical to *AvrPii* (Genbank accession no.: AB498874).

**Table 2:** Haplotypes of 5 avirulence (*Avr*) genes in 27 isolates. +: Identical to the respective Avr gene; −: Do not contain the respective Avr gene; LTR: long terminal repeat

SEQ	Name of the strains	*AvrPik*					*AvrPii*	*AvrPi9*	*AvrPiz-t*		*AvrPib*	
		Amino acid position				Haplotype			Type of mutations and transposons	Haplotype	Type of mutations and transposons	Haplotype
		46	47	48	67							
Strain1	08-T19	N	P	G	A	*AvrPik-E*	–	+	Inago2 LTR retrotransposon; − 182 bp	*AvrPiz-t-H3*	1.8 kb pot3 transposon; + 170 bp	*AvrPib-H2*
Strain2	08-T29	H/N	P/A	G/D	A	*AvrPik-A/D*	–	+	+	*AvrPiz-t-H1*	1.8 kb pot3 transposon; −125 bp	*AvrPib-H8*
Strain3	10–157	N	P	G	A	*AvrPik-E*	–	+	1 SNP[41th amino acid GCG(A) to GTG(V)]	*AvrPiz-t-H2*	495 bp pot3 transposon; −71 bp	*AvrPib-H9*
Strain4	10–402	N	P	G	A	*AvrPik-E*	–	+	+	*AvrPiz-t-H1*	+	*AvrPib-H1*
Strain5	10–431	H/N	P	G	A	*AvrPik-D/E*	–	+	+	*AvrPiz-t-H1*	Deletion at positions 37 and 38 bp	*AvrPib-H4*
Strain6	10–432	N	P	G	A	*AvrPik-E*	–	+	+	*AvrPiz-t-H1*	495 bp pot3 transposon; −71 bp	*AvrPib-H9*
Strain7	10–555	H/N	P	G	A	*AvrPik-D/E*	–	+	+	*AvrPiz-t-H1*	Deletion at positions 37 and 38	*AvrPib-H4*
Strain8	10–649	N	P	G	A	*AvrPik-E*	–	+	+	*AvrPiz-t-H1*	1.8 kb pot3 transposon; − 274 bp	*AvrPib-H6*
Strain9	11–13	N	P	G	A	*AvrPik-E*	–	+	+	*AvrPiz-t-H1*	1.8 kb pot3 transposon; −274 bp	*AvrPib-H6*
Strain10	11–121					–	–	+	+	*AvrPiz-t-H1*	1.8 kb pot3 transposon; − 274 bp	*AvrPib-H6*
Strain11	11–239	N	P	G	A	*AvrPik-E*	–	+	+	*AvrPiz-t-H1*	+	*AvrPib-H1*
Strain12	11–445	H/N	P	G	A	*AvrPik-D/E*	–	+	Inago2 LTR retrotransposon; −182 bp	*AvrPiz-t-H3*	1.8 kb pot3 transposon; −56 bp	*AvrPib-H5*
Strain13	11–882	N	P	G	A	*AvrPik-E*	–	+	+	*AvrPiz-t-H1*	1.8 kb pot3 transposon; − 56 bp	*AvrPib-H5*
Strain14	11–909	N	P	G	A	*AvrPik-E*	–	+	+	*AvrPiz-t-H1*	1.8 kb pot3 transposon; −274 bp	*AvrPib-H6*
Strain15	11–1093	N	P	G	A	*AvrPik-E*	–	+	+	*AvrPiz-t-H1*	1.8 kb pot3 transposon; −274 bp	*AvrPib-H6*
Strain16	12–3055	H/N	P	G	A	*AvrPik-D/E*	–	+	Inago2 LTR retrotransposon; −182 bp	*AvrPiz-t-H3*	1.8 kb pot3 transposon; −125 bp	*AvrPib-H8*
Strain17	12–3057	H/N	P	G	A	*AvrPik-D/E*	–	+	Inago2 LTR retrotransposon; −182 bp	*AvrPiz-t-H3*	1.8 kb pot3 transposon; −65 bp	*AvrPib-H7*
Strain18	13–123	H/N	P	G	A	*AvrPik-D/E*	–	+	+	*AvrPiz-t-H1*	Deletion at positions 37 and 38 bp	*AvrPib-H4*
Strain19	13–227	H/N	P	G	A	*AvrPik-D/E*	–	+	Inago2 LTR retrotransposon; −182 bp	*AvrPiz-t-H3*	1.8 kb pot3 transposon; −56 bp	*AvrPib-H5*
Strain20	13–412	H/N	P	G	A	*AvrPik-D/E*	–	+	Inago2 LTR retrotransposon; −182 bp	*AvrPiz-t-H3*	1.8 kb pot3 transposon; −56 bp	*AvrPib-H5*
Strain21	13–466	H/N	P	G	A	*AvrPik-D/E*	–	+	Inago2 LTR retrotransposon; −182 bp	*AvrPiz-t-H3*	1.8 kb pot3 transposon; −65 bp	*AvrPib-H7*
Strain22	13–594					–	–	+	+	*AvrPiz-t-H1*	+	*AvrPib-H1*
Strain23	13–710	H/N	P	G	A	*AvrPik-D/E*	–	+	+	*AvrPiz-t-H1*	Deletion at positions 37 and 38 bp	*AvrPib-H4*
Strain24	93–286	N	P	G	A	*AvrPik-E*	–	+	+	*AvrPiz-t-H1*	495 bp pot3 transposon; −71 bp	*AvrPib-H9*
Strain25	98–288	H/N	P	G	A	*AvrPik-D/E*	+	+	+	*AvrPiz-t-H1*	1.8 kb pot3 transposon; −125 bp	*AvrPib-H8*
Strain26	w08–59	H	P	G	A	*AvrPik-D*	–	+	+	*AvrPiz-t-H1*	A97T	*AvrPib-H3*
Strain27	00–193	H/N	P	G	A	*AvrPik-D/E*	–	+	+	*AvrPiz-t-H1*	A97T	*AvrPib-H3*

*AvrPik*: 3 haplotypes corresponded to *AvrPik*-*A*, −*D*, and –*E* (Genbank accession nos.: AB498875-AB498879). Fourteen isolates contained *AvrPik-D* (Table 2). Intriguingly, out of these 14 isolates, 12 contained *AvrPik*-*E* and 1 contained *AvrPik*-*A*, suggesting that most of *AvrPik*-*D* containing isolates had an additional *AvrPik* haplotype. On the contrary, 11 isolates contained only *AvrPik-E* (Table 2). Taken together, *AvrPik-E* was identified in 23 isolates, representative of an approximately frequency of 85%. Two isolates were unable to be resolved with any PCR amplicon (Table 2). Pathogenicity of isolates containing *AvrPik-D*, −*E*, and -*A* to *Pi1*-, *Pikh*-, and *Pikp*-containing monogenic lines revealed that *AvrPik-E* and -*A* depleted the resistance mediated by *Pikp* (Additional file 6: Table S4), which is consistent with previous findings (Chaipanya et al. 2017; Selisana et al. 2017).

*AvrPi9*: It was found in all isolates and amplicon sequencing further revealed that it was identical to *AvrPi9* (Genbank accession no.: KM004023) (Table 2).

*AvrPiz-t*: 3 haplotypes corresponded to *AvrPiz-t*-*H1* ~ *AvrPiz-tH3* (Table 2). *AvrPiz-t-H1* identical to *AvrPiz-t* (Genbank accession no.: EU837058) was identified in 19 isolates representative of an approximately 70% of frequency (Table 2). *AvrPiz-t-H2* was identified only in the isolate 10–157 and encoded for AvrPiz-t^A4IV^, caused by a single nucleotide substitution from C to T which corresponded to the 41st residue as described previously (Li et al. 2009). *AvrPiz-t-H3* was found in 7 isolates which contained an approximately 6-kb insertion deduced by PCR amplification using a pair of primers in the promoter of *AvrPiz-t* (AvrPizt-300F/AvrPizt-10R). Amplicon sequencing of this PCR amplicon further revealed that it was an Inago 2-like retrotransposon [98% sequence similarity to the published one (Genbank accession no.: AB334125), which was inserted at the position of -182 bp with a 5-bp “AATGC” target site duplication (Table 2). Besides the insertion of an Inago 2-like element, no sequence difference was found from *AvrPiz-t*. Both *AvrPiz-t-H2* and *AvrPiz-t*-*H3* were deduced to be virulent to *Piz-t* based on their pathogenicity to *Piz-t* monogenic line (Additional file 6: Table S4).

*AvrPib*: 9 different haplotypes designated as *AvrPib-H1* ~ *AvrPib-H9* were identified based on the sequences of PCR amplicons (Table 2). *AvrPib-H1* was identical to *AvrPib* (Genbank accession no.: KM887844) and identified in only 3 isolates. *AvrPib-H2*, *AvrPib*-*H4* and *AvrPib-H9* were identical to those described previously (Zhang et al. 2015). However, *AvrPib-H3* contained a single nucleotide substitution from A to T at the position of + 97 (Genbank accession no.: MN630588), and *AvrPib-H5* ~ *AvrPib-H8* each contained a Pot3 element at − 56, − 274, − 65, − 125 bp, respectively, based on the *AvrPib* sequence (Table 2). All the *AvrPib* haplotypes except *AvrPib-H1* were deduced to be virulent to *Pib* based on their pathogenicity to *Pib* monogenic line (Additional file 6: Table S4).

### Resistance Spectrum Analysis of 54 Rice Varieties to Differential Isolates

**Fig. 2:**
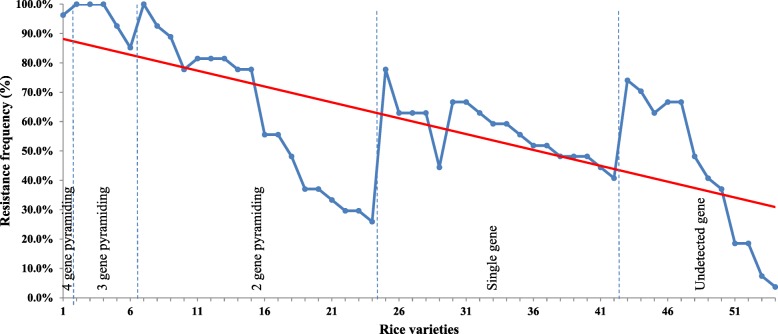
Resistance frequency of 54 rice varieties. Rice varieties are listed in the order of number of pyramided *R* genes as in Additional file 6: Table S4. The number of pyramided *R* genes is indicated by lines. The regression curve is drawn along the resistance frequency

The aforementioned 27 differential isolates were used to inoculate all 54 rice varieties at the seedling stage to determine their resistance spectra. A range from 3.7% to 100% of resistance frequencies (RFs) was observed in these 54 rice varieties, indicating that they differed significantly in the resistance to the same rice blast population (Additional file 6: Table S4). Three varieties (Chuanyouhuazhan, Wuyouhuazhan, and Xinrongyouhuazhan) were resistant to all isolates whereas Eyi105 and Ewan were resistant to less than 10% of isolates (Additional file 6: Table S4). It was noted that 23 rice varieties contained multiple known *R* genes, indicating that *R* genes were stacked in many varieties (Table 1 and Fig. 2). Among them, 6 varieties contained 3 or 4 *R* genes and were highly resistant to the differential isolates (Additional file 6: Table S4 and Fig. 2). It is worth noting that 5 of them, i.e., Tianyouhuazhan, Wuyouhuazhan, C-liangyouhuazhan, Xinrongyouhuazhan, and Rongyouhuazhan, were derived from the same male parental line, Huazhan and all contained *Pi2*, *Pi5*, and *Pib* (Additional file 1: Table S1 and Additional file 6: Tables S4). It is thus reasonable to speculate that the restore line Huazhan could contain all or some of these 3 *R* genes. A relatively positive correlation between resistance and the number of stacked *R* genes in the varieties was observed based on the regression analysis (Fig. 2), suggesting that the pyramiding of multiple *R* genes rendered the high level of resistance of varieties to the rice blast isolates.

To deduce the contribution of different *R* genes to the varietal resistance, we included nine monogenic lines including 9 different IRBLs and NIL-e1 for *Pi50* in the assay. Different monogenic lines displayed varied RFs ranging from 3.7% to 100% to the differential isolates (Additional file 6: Table S4). The *Pi9* monogenic line was resistant to all isolates whereas *Pii* monogenic line is resistant to only one isolate (Additional file 6: Table S4). RFs of 7 monogenic lines (IRBL9-W, IRBLzt-T, IRBLkh-K3, IRBL1-CL, IRBLkp-K60, IRBLi-F5, and IRBLb-B) evaluated by pathogenicity tests had the same value of the frequency of respective cognate *Avr* genes in 27 differential isolates (Table 2 and Additional file 6: Table S4). These results reiterated the reliability of the *Avr*-gene based diagnosis for determining the isolates’ pathotypes to respective *R* genes (Selisana et al. 2017). It is worth noting that *Pikh* and *Pi1* isogenic lines showed the same RF (93%), which is much higher than the one of *Pikp* (52%). The discrepancy of the RF between *Pikp* and *Pikh*/*Pi1* is attributable to the presence of 41% of isolates containing only *AvrPik-E* which is able to defeat *Pikp* (Table 2). The result herein verified again the distinct recognition specificities between different *Pik* and *AvrPik* alleles (Kanzaki et al. 2012; Selisana et al. 2017). It is noted that the RFs of some monogenic lines were used to infer those of other *R*-gene alleles i.e., *Pikp*, *Pi1*, and *Pii* for *Pi7*, *Pikm*, and *Pi5*, respectively, since they controlled the same resistance spectra as described previously (Selisana et al. 2017). Based on the RF of each known *R* gene at the targeted 4 *R*-gene loci, the contribution rates of known *R* gene(s) to the varieties were deduced. The interaction pattern of the stacked *R* genes resulted in distinct effects on the RFs of varieties. For example, the resistance conferred by *Pib* was perfectly masked by the one controlled by *Pi2*, leading to no additive effect on RFs of rice varieties containing both of them. On the contrary, no overlapped resistance was identified between *Pi7* and *Pib*, leading to completely additive effects of RFs of rice varieties stacked with both of them (Additional file 6: Table S4).

### The Contribution of *R* Genes to the Resistance of Rice Populations

**Fig. 3:**
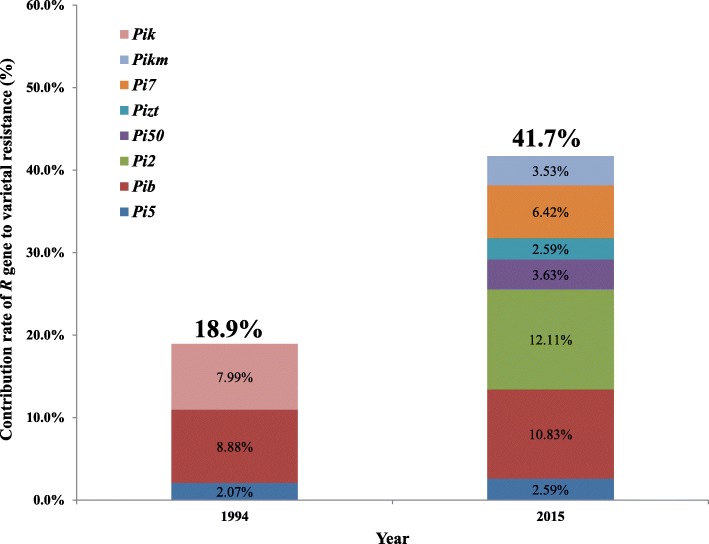
Contribution of different *R* genes to the resistance of varieties in 1994 and 2015. The percentages of individual *R* genes contributed to the varietal resistance are indicated in colored boxes corresponding to the different *R* genes. The accrued percentage from individual *R* genes is indicated on the top of boxes for the years of 1994 and 2015

To evaluate the contribution of *R* genes to rice resistance at the population level, we calculated the accumulative effects of individual *R* genes to the resistance of 54 rice varieties against 27 isolates. The total number of resistance reactions attributable to the individual *R* genes was then summed. The ratio of the summed value against the total reactions was then calculated and represented as the contribution rate of the respective *R* gene to the rice population. As Fig. 3 illustrated, around 18.9% of accumulative resistance controlled by three *R* genes, i.e., *Pik*, *Pib*, and *Pi5*, was identified in the population of 1994. However, around 41.7% of resistance conferred by 7 *R* genes was identified in the population of 2015, indicating that rice varieties in 2015 were overall more resistant than those in 1994. Both *Pib* and *Pi2* contributed more significantly to the resistance than other *R* genes. Nevertheless, it is worth noting that the larger contribution of *Pib* to the resistance was partly because of the higher frequency of *Pib* in rice varieties (Table 1 and Fig. 1). We also measured the percentile of rice varieties resistant to more than 50% of isolates in both populations of 1994 and 2015. It is evident that more varieties in 2015 showed no lower than 50% resistance to the isolates than in 1994 (67.5% versus 50%) (Additional file 7: Figure S3). These data indicated that varieties in 2015 showed a higher level of resistance than those in 1994 to rice blast.

We further classified the resistant and susceptible reactions to 4 different categories depending on the presence or absence of known *R* genes. Category I represented the resistant reaction with the presence of known *R* gene(s); category II represented the susceptible reaction with the presence of known *R* gene(s); category III represented the resistant reaction without the presence of known *R* gene(s); category IV represented the susceptible reaction without the presence of known *R* gene(s) (Additional file 8: Figure S4). The number of reactions sitting in different categories was summed separately. Around 21.5% of total reactions belonged to category I, demonstrating that the presence of known *R* gene(s) leads to the varietal resistance against rice blast (Additional file 8: Figure S4). Consistently, around 12.9% of reactions belonging to category IV illustrated another expected outcome in which varieties without known *R* genes were susceptible. On the contrary, around 26.6% of reactions belonging to category II were susceptible although the varieties contained known *R* genes, indicating that these *R* genes eroded in their function already (Additional file 8: Figure S4). Category III represented another outcome in which varieties showed resistance although no known *R* genes were identified. We postulated that most likely some unknown *R* genes were attributable to the varietal resistance in this category. It is worth noting that the percentile of category III is much higher than the one of category I (39% versus 21.5%), indicating that unknown *R* genes rather than known *R* genes contributed more significantly to the resistance of varieties used in this study (Additional file 8: Figure S4). It was also found that only 14 out of 54 rice varieties in which known *R* gene(s) contributed not less than 50% of resistance (Additional file 6: Table S4).

## Discussions

Resistance to rice blast to a certain level is one of indispensable requirements for varietal releasing in the vetting system in some rice growing countries, such as China (Zeng et al. 2017; Pandian et al. 2018). Therefore, rice blast resistance has been considered as one of the priorities in the rice breeding program. *R*-gene linked or specific molecular markers were developed and widely used in the mainstream breeding programs via marker aided selection (MAS) (Jiang et al. 2012; Balachiranjeevi et al. 2015; Ni et al. 2015; Ji et al. 2016; Shalini et al. 2016; Wu et al. 2016; Mi et al. 2018). However, many rice blast *R*-gene haplotypes from different rice lines are almost identical except for a few nucleotide differences, leading to different specificities or even loss of function, *e.g*, different *Pik* and *Pi2/9* alleles (Zhou et al. 2007; Constanzo and Jia 2010). In this regard, it is critical to develop markers with perfect correlation with their targeted *R* genes for the diagnosis and selection. A most recent study documented the frequencies of different blast *R* genes in 161 Indian rice landraces using *R*-gene linked markers (Yadav et al. 2019). In this study, approximately 12.42% and 85% of landraces contained *Pi2* and *Piz-t*, respectively. Moreover, the landraces containing *Piz-t* were surprisingly noted to be positive for *Piz* (Yadav et al. 2019). Contrasting to the extremely high frequency of *Pi2/9* alleles described above, only 1 and 5 lines were identified to contain *Pi2* and *Piz-t* out of 1400 rice landraces by referring to the complete sequence of coding region, suggesting a very low frequency of functional *Pi2/9* alleles was inherited in rice landraces (Xiao et al. 2017). This contrasting distribution pattern of different alleles at the same *R* gene locus could be explained to be attributable to different rice population used. Nevertheless, it is necessary to validate the data interpretation by gene sequencing rather than merely by PCR amplification. In this study, we employed a similar method of amplicon sequencing as described previously (Xiao et al. 2017) and applied it for the diagnosis of 4 *R*-gene loci in a selected set of popular rice varieties particularly hybrids released in the past two decades in China. The fragment amplified and sequenced covered the region mainly determining the specificities of different alleles, capable for distinguishing different alleles from one another. In addition, novel alleles containing sequence variations can also be classified, providing a de novo approach for discovering new variants in addition to diagnosis of known ones. As an example for the haplotypes at the *Pi2/9* locus, we identified 1 and 6 rice lines contained *Piz-t* and *Pi2*, respectively, suggesting that either *Piz-t* or *Pi2* was not frequently introduced into the elite rice varieties although they were cloned over 10 years ago (Zhou et al. 2006). A similar finding in which the *Pi2* gene was not detected in all 88 elite hybrid rice varieties in China using the gene specific marker was reported recently (Wu et al. 2017). In addition to *Pi2* and *Piz-t*, a novel *Pi2/9* allele *Pi2/9-V20* most likely not responsible for the varietal resistance was identified in a significant number of rice lines, providing a clue for tracing its inheritance in different rice lines. It is worth noting that the current approach is based on the traditional PCR amplification and subsequent sequencing using Sanger method, which is relatively low throughput. Moreover, we note that partial sequence analysis of *R*-gene haplotypes may not be sufficient to provide accurate diagnosis of blast *R* genes due to presence of mutations in the un-sequenced regions. However, we would like to insist that comparison of the sequences corresponding to the critical portion of *R*-gene haplotypes is at least able to differentiate them from known sequences. Furthermore, we focused the analysis in the released varieties rather than in diverse germplasm. A high probability for the deduction by referring to the core portion of known *R*-gene haplotypes is thus expected since the same *R*-gene donor is often used for breeding the resistant varieties. Recently, a more robust approach so called targeted amplicon sequencing (TAS) using the next-generation sequencing platform was introduced and optimized for a higher scalability and cost efficient platform for the identification of variants of targeted genes (Meryer et al. 2007; Pertoldi et al. 2009; Bybee et al. 2011). The customized TAS technology provided by companies, e.g., illumina®, enables researchers to efficiently discover and screen genetic variants in hundreds to thousands samples. The future development of diagnostic system using the TAS technology for both *R* and *Avr* genes in the rice and rice blast phyto-pathosystem will be promising for implementing a high-throughput analysis applicable for the field monitoring.

The continuous deployment of resistant variety containing same *R* gene(s) is believed to erode its resistance soon or later, which is speculated to be attributable to the emergence and subsequent dominancy of virulent isolates in the population (Valent and Khang 2010; Wang et al. 2017). In addition to this temporal effect, the spatial effect resulted from the wide distribution of the *R* genes in different varieties could further increase the risk of defeat of resistance. As reported in this study, *Pib* and *Pi5,* which are widely distributed in rice varieties, do not contribute significantly to the varietal resistance any longer. Consistently, the frequencies of their cognate *Avr* genes in the rice blast population are relatively low, which is most likely attributable to the constant selection pressure posed by the varieties with large planting areas. On the contrary, those *R* genes, which are yet deployed widely in rice varieties, show relatively strong resistance to the disease, such as *Pikm*, *Pi50*, and *Pi9*. The correlation between the wide distribution of *R* genes and relatively low resistance frequency conferred by them documented in this study might need more case studies for the further validation. However, the suicide effect of a particular *R* gene caused by its wide deployment in mega varieties raises a caution for the deployment of *R* genes. It is worth noting that *Pi2* showed a very high frequency of resistance against a rice blast population consisting of 792 isolates, suggesting it was one of promising *R* genes conferring broad-spectrum resistance against rice blast (Chen et al. 1999). The identification and cloning expedited the use of *Pi2* in different rice varieties using MAS (Zhou et al. 2006; Liu et al. 2008; Mi et al. 2018). As of February 2019, Huazhan harboring *Pi2* was used as the parental line for a total of 123 hybrid rice varieties, ranking the top restorer line for the breeding of the most number of hybrids (http://www.ricedata.cn/). Due to the highly genetic instability of rice blast pathogen, the efficiency of deployed rice blast *R* genes in genetically uniform monocultures was speculated to decline continuously over time (Mcdonald and Linde 2002). The finding that the resistance frequency of *Pi2* was relatively lower than *Piz-t* and *Pi50* as reported in this study does imply a risk of the breakdown of *Pi2* resistance especially along the quick spreading of Huazhan derived rice varieties.

The finding that quite a high frequency of the same non-functional allele at some *R*-gene loci, such as *Pikm1-LTH*, *Pib-GII*, and *Pi2/9-V20* respectively at *Pik*, *Pib*, and *Pi2/9* loci, is surprising and cautious to some degree. It indicated that the genetic diversity of *R* genes in modern rice varieties is not only limited but also void for resistance. Moreover, it implied that the existing breeding method is cost inefficient and less effective in improving blast resistance since the non-functional haplotypes of the *R*-gene loci have been unintentionally introgressed and spread in different rice varieties. It is elusive how these non-functional *R*-gene haplotypes were introduced and retained in the elite rice varieties. However, the selection method for the truly resistant breeding lines including phenotyping by resistance assessment and genotyping using molecular markers could be revisited and refined, which will likely improve the selection efficiency and reliability.

## Conclusions

Our study provided an extensive sequence-based diagnosis of *R*-gene haplotypes in a set of elite rice varieties. The results revealed that quite a number of *R*-gene haplotypes was non-functional, suggesting that desired *R*-gene haplotypes were not successfully incorporated in elite rice varieties. Moreover, some *R*-gene haplotypes were defeated of their resistance due to the low frequency of their cognate *Avr* genes or haplotypes. Our study envisages an increasing demand on the introgression of effective *R* genes in rice breeding program through an intensive *R* gene specific marker selection. We anticipate that the sequence-based diagnosis as described in this study could be widely adopted for validating the presence of rice blast *R* genes in elite rice varieties and diverse germplasm as well.

## Supplementary information

**Additional file 1: Table S1.** Rice accessions used in this study.

**Additional file 2: Table S2.** *M. oryzae* isolates used in this study.

**Additional file 3: Table S3.** Primers used in this study.

**Additional file 4: Figure S1.** Validation of *R*-gene specific primers for PCR amplification.

**Additional file 5: Figure S2.** Disproportional distribution of different known *R* genes in rice varieties.

**Additional file 6: Table S4.** Resistance assessment of 54 selected rice varieties against 27 rice blast isolates.

**Additional file 7: Figure S3.** Resistance frequency of varieties in 1994 (A) and 2015 (B).

**Additional file 8: Figure S4.** Deduction of contribution of known and unknown *R* genes to the rice varieties.

## Data Availability

All data generated or analyzed during this study are included in this published article and its supplementary information files.

## Abbreviations

*Avr* genes: Avirulence genes
HMA: Heavy metal-associated
HR: Highly resistant
HS: Highly susceptible
IRBLs: IRRI bred blast resistant lines
IRRI: International rice research institute
LRR: Leucine-rich repeat
LTH: Lijiangxintuanheigu
MAS: Marker assisted selection
MR: Moderately resistant
MS: Moderately susceptible
NBS: Nuclear binding site
NCBI: National center for biotechnology information
NIL-e1: Near isogenic line
PCR: Polymerase chain reaction
*R* genes: Resistance genes
R: Resistant
RFs: Resistance frequencies
RGA: Resistance gene analog
S: Susceptible
TAS: Targeted amplicon sequencing
