# Supplementary material for: Prevalence of Ineffective Haplotypes at the Rice Blast Resistance (R) Gene Loci in Chinese Elite Hybrid Rice Varieties Revealed by Sequence-Based Molecular Diagnosis
Source: Rice (N Y). 2020 Jan 30;13:6. doi: 10.1186/s12284-020-0367-x (PMC6990218; doi:10.1186/s12284-020-0367-x)
Supplement: Supplementary file 1 — Additional file 1: Table S1. Rice accessions used in this study. [file 12284_2020_367_MOESM1_ESM.docx]

Table S1. Rice accessions used in this study

| Accessions | Classification | Year | Promotion area (×10^4^ ha) | Female | Male |
| --- | --- | --- | --- | --- | --- |
| Shanyou 63 | Hybrid | 1994 | 447.761 | Zhenshan 97A | Minghui 63 |
| Zhefu 802 | Inbrid | 1994 | 38.86 | Simei No.2 | |
| Zhe 773 | Inbrid | 1994 | 37.587 | Hezhenzao | Chikuaiaixuan |
| Wuyugeng No.3 | Inbrid | 1994 | 36.448 | 79-51 | Yanggeng No.1 |
| Shanyou 46 | Hybrid | 1994 | 32.495 | Zhenshan 97A | Miyang 46 |
| Gengxian 89 | Hybrid | 1994 | 31.289 | IR36 | Gengxian677 |
| Weiyou 46 | Hybrid | 1994 | 29.279 | Wei 20A | Miyang 46 |
| Weiyou 77 | Hybrid | 1994 | 25.862 | Wei 20A | Minghui 77 |
| Wuyugeng No.2 | Inbrid | 1994 | 25.661 | 79-51 | Yanggeng No.1 |
| Qishanzhan | Inbrid | 1994 | 19.095 | Qiguizao25 | Guishanzao |
| Xiangzaoxian No.7 | Inbrid | 1994 | 17.755 | 81-280 | HA79317-4 |
| Ewan No.5 | Inbrid | 1994 | 15.611 | Ewan No.3 | 4243 |
| Eyi 105 | Inbrid | 1994 | 11.256 | Nongken58 | |
| Xiushui 122 | Inbrid | 1994 | 9.447 | Xiushui04 | Xiushui620 |
| Shenliangyou 5814 | Hybrid | 2015 | 26.867 | Y58S | B4114 |
| Y liangyou No.1 | Hybrid | 2015 | 26.867 | Y58S | 9311 |
| Wuyou 308 | Hybrid | 2015 | 23.919 | Wufeng A | Guanghui 308 |
| Tianyouhuazhan | Hybrid | 2015 | 23.785 | Tianfeng A | Huazhan |
| Yangliangyou No.6 | Hybrid | 2015 | 15.745 | Guangzhan 63-4S | Yangdao No.6 |
| Chuanyou 6203 | Hybrid | 2015 | 14.472 | Chuang106A | Chenhui3203 |
| Gangyou 188 | Hybrid | 2015 | 14.204 | Gang46A | Lehui188 |
| Wuyouhuazhan | Hybrid | 2015 | 11.256 | Wufeng A | Huazhan |
| C liangyouhuazhan | Hybrid | 2015 | 11.122 | C815S | Huazhan |
| Xinliangyou No.6 | Hybrid | 2015 | 10.921 | Xinan S | Anxuan No.6 |
| Y liangyou 5867 | Hybrid | 2015 | 10.72 | Y58S | R674 |
| Zhongzheyou No.8 | Hybrid | 2015 | 10.653 | Zhongzhe A | T-8 |
| Zhongzheyou No.1 | Hybrid | 2015 | 9.916 | Zhongzhe A | Hanghui570 |
| Xinrongyouhuazhan | Hybrid | 2015 | 9.581 | Xinrong A | Huazhan |
| Yueyou 9113 | Hybrid | 2015 | 9.313 | Yue 4A | Yuehui 9113 |
| Rongyou 225 | Hybrid | 2015 | 9.045 | Rongfeng A | R225 |
| Rongyouhuazhan | Hybrid | 2015 | 8.911 | Rongfeng A | Huazhan |
| Liangyou 6326 | Hybrid | 2015 | 8.844 | Xuan69S | Zhongxian Wh26 |
| H you 518 | Hybrid | 2015 | 8.777 | H28A | 51084 |
| Luliangyou 996 | Hybrid | 2015 | 8.576 | Lu18S | 996 |
| Guangliangyouxiang 66 | Hybrid | 2015 | 8.174 | Guangzhan 63-4S | Xianghui 66 |
| C liangyou 396 | Hybrid | 2015 | 8.107 | C815S | R396 |
| Wandao153 | Hybrid | 2015 | 7.973 | 1891S | RH003 |
| Fengliangyou No.4 | Hybrid | 2015 | 7.839 | Feng39S | Yandao No.4 Xuan |
| F you 498 | Hybrid | 2015 | 7.772 | FS3A | Shuhui 498 |
| C liangyou 343 | Hybrid | 2015 | 7.437 | C815S | Yuehui 9113 |
| Yueyou518 | Hybrid | 2015 | 7.303 | Yue 4A | R518 |
| Wufengyou T025 | Hybrid | 2015 | 7.236 | WufengA | changhui T025 |
| Taiyou 390 | Hybrid | 2015 | 7.236 | TaifengA | Guanghui 390 |
| Tanliangyou83 | Hybrid | 2015 | 6.834 | Tannong S | Tanzao 183 |
| Tianfengyou 316 | Hybrid | 2015 | 6.365 | TianfengA | Chanhui316 |
| Y liangyou 9918 | Hybrid | 2015 | 6.03 | Y58S | R928 |
| C liangyou 608 | Hybrid | 2015 | 5.963 | C815S | R608 |
| Liangyou 688 | Hybrid | 2015 | 5.963 | SE21S | Nanhui688 |
| Huiliangyou 996 | Hybrid | 2015 | 5.695 | 1892S | R996 |
| Tianyou 998 | Hybrid | 2015 | 5.628 | TianfengA | Guanghui 998 |
| Y liangyou No.6 | Hybrid | 2015 | 5.226 | Y58S | Wanghui006 |
| Zhuliangyou 819 | Hybrid | 2015 | 5.226 | Zhu 1S | Hua 819 |
| Fengyuanyou 299 | Hybrid | 2015 | 5.159 | FengyuanA | Xianghui 299 |
| Dexiang 4103 | Hybrid | 2015 | 5.159 | Dexiang074A | Luhui H103 |
