# Supplementary material for: Prevalence of Ineffective Haplotypes at the Rice Blast Resistance (R) Gene Loci in Chinese Elite Hybrid Rice Varieties Revealed by Sequence-Based Molecular Diagnosis
Source: Rice (N Y). 2020 Jan 30;13:6. doi: 10.1186/s12284-020-0367-x (PMC6990218; doi:10.1186/s12284-020-0367-x)
Supplement: Supplementary file 2 — Additional file 2: Table S2. M. oryzae isolates used in this study. [file 12284_2020_367_MOESM2_ESM.docx]

Table S2. *M. oryzae* isolates used in this study. The name, year, place and variety of collection of the isolate were list in the table.

| Seq | Isolate | Year | Collection place | Variety |
| --- | --- | --- | --- | --- |
| Strain1 | 08-T19 | 2008 | Longma village, songzhu town, leizhou city, zhanjiang, guangdong province | Tianyou 998 |
| Strain2 | 08-T29 | 2008 | Longma village, songzhu town, leizhou city, zhanjiang, guangdong province | Texianzhan 25 |
| Strain3 | 10-157 | 2010 | Sanjiao town, liangping county, heyuan city, guangdong province | Fengyouzhan |
| Strain4 | 10-402 | 2010 | Shiqiao village, tieyong town, huidong county, huizhou city, guangdong province | Yingzhan |
| Strain5 | 10-431 | 2010 | Lianpeng village, minshan town, huidong county, huizhou city, guangdong province | Fengsizhan |
| Strain6 | 10-432 | 2010 | Lianpeng village, minshan town, huidong county, huizhou city, guangdong province | Yingzhenzhan |
| Strain7 | 10-555 | 2010 | Nanpu, longxian town, wengyuan county, shaoguan city, guangdong province | GDBL9 |
| Strain8 | 10-649 | 2010 | Lu tian, conghua district, guangzhou city, guangdong province | Meixiangzhan |
| Strain9 | 11-13 | 2011 | Luokeng town, qujiang district, shaoguan city, guangdong province | Mabayingzhan |
| Strain10 | 11-121 | 2011 | Jiuhe town, zijin county, heyuan city, guangdong province | Tianyou 122 |
| Strain11 | 11-239 | 2011 | Xibi village, qichong town, lufeng city, shanwei, guangdong province | Zhenguiai |
| Strain12 | 11-445 | 2011 | Tangping town, yangdong county, yangjiang city, guangdong province | Shuangyou8802 |
| Strain13 | 11-882 | 2011 | Ao feng village, bomei town, lufeng city, shanwei, guangdong province | Meiyou 998 |
| Strain14 | 11-909 | 2011 | Nanfeng town, fengkai county, zhaoqing city, guangdong province | Qiuyou 998 |
| Strain15 | 11-1093 | 2011 | Huangnitian Canton, langlian village, magui town, gaozhou city, maoming city, guangdong province | Jianyou 381 |
| Strain16 | 12-3055 | 2012 | Longchuan county, heyuan city, guangdong province | Yuejingsimiao No.2 |
| Strain17 | 12-3057 | 2012 | Longchuan county, heyuan city, guangdong province | Yuejingsimiao No.2 |
| Strain18 | 13-123 | 2013 | Wushishigui village, zijin county, heyuan city, guangdong province | Unknown |
| Strain19 | 13-227 | 2013 | Tanyun village, huaiji town, zhaoqing city, guangdong province | Teyou 9846 |
| Strain20 | 13-412 | 2013 | Banxi village, ba zi town, wengyuan county, shaoguan city, guangdong province | Tianyou 363 |
| Strain21 | 13-466 | 2013 | Agricultural science institute of longchuan county, heyuan city, guangdong province | Yuejingsimiao No.2 |
| Strain22 | 13-594 | 2013 | Blast nursery of Baisha, yangjiang city, guangdong province | L4 |
| Strain23 | 13-710 | 2013 | Jintang village, deyao village, gaoqiao town, lianjiang city, zhanjiang city, guangdong province | Ylaingyou No.1 |
| Strain24 | 93-286 | 1993 | Guigang town, yangchun city, yangjiang city, guangdong province | Qingyou 159 |
| Strain25 | 98-288 | 1998 | Zhaoqing city, guangdong province | Fengbazhan |
| Strain26 | w08-59 | 2008 | Blast nursery of enshi,Hubei province | Unknown |
| Strain27 | 00-193 | 2000 | Haifeng county, shanwei city, guangdong province | Zhenguiai |
