# Supplementary material for: Prevalence of Ineffective Haplotypes at the Rice Blast Resistance (R) Gene Loci in Chinese Elite Hybrid Rice Varieties Revealed by Sequence-Based Molecular Diagnosis
Source: Rice (N Y). 2020 Jan 30;13:6. doi: 10.1186/s12284-020-0367-x (PMC6990218; doi:10.1186/s12284-020-0367-x)
Supplement: Supplementary file 3 — Additional file 3: Table S3. Primers used in this study. [file 12284_2020_367_MOESM3_ESM.docx]

Table S3. Primers used in this study. *R*: resistance; *Avr*: avirulence

| Gene/ Locus | Primer name | Primer sequence (5'to 3') | Expected Size  (bp) | Reference |
| --- | --- | --- | --- | --- |
| *R* gene primer | | | | |
| *Pi2/9* | Pi2/9-DF1 | CTTGACATCCAAACCGCACC | 1172 | Xiao et al. 2017 |
|  | Pi2/9-DR1 | TAGGCCTAGCCAATTTTTGCC |  |  |
|  | Pi2/9-F3 | AGTTGTTTGCACATGGTGCTGGATG | 3878 |  |
|  | Pi2/9-R4 | TCAGCCAGCTTGAGCTGTGCCTATC |  |  |
| *Pib* | Pib-F4 | CGTGATGCGTGGACTTTCGC | 915 | Olukayode et al. 2018 |
|  | Pib-R7 | TAACTCCAAAGGAGCTCAGG |  |  |
| *Pik* | RGA4F3 | GGAAAGCTGATATGTTGTCG | 1326 |  |
|  | RGA4R3 | ACTCGGAGTCGGAGAGTCAG |  |  |
| *Pi5* | 09RL09 F2 | TCTGGTGAAAACTCTGAACG | 740 |  |
|  | 09RL09 R2 | CGGTGAGTTTAGCGAAGACC |  |  |
| *Avr* gene primer | | | | |
| *AvrPi9* | AvrPi9-CDSF | AGGATTCCAGCTATTCGAC | 624 |  |
|  | AvrPi9-CDSR | CAGTAATGCGCAGAAGGGTGT |  |  |
| *AvrPizt* | AvrPizt-F | GTTGCGATTATGATCCGTCG | 1144 |  |
|  | AvrPizt-R | GTACTCTAGCAAACGACCGG |  |  |
|  | AvrPizt-300F | TTCCATCTCACCAATGTTGG | 352 |  |
|  | AvrPizt-10R | TGGATTTGATGTCAGCGATG |  |  |
| *AvrPib* | 10583F | GAAGACTTTGATGCAAATGG | 597 | Olukayode et al. 2018 |
|  | 10583R | GAGAATGCTAGCTAGATCTG |  |  |
| *AvrPik* | AvrPik-F | TCCTGCTGCTAACTCCATTC | 1200 |  |
|  | AvrPik-R | TCAACCAAGCGTAAACCTCG |  |  |
| *AvrPii* | AvrPii-F | GGTAGATATCCGCTGACTGG | 840 |  |
|  | AvrPii-R | ACTGTCCGCCGCTCGTTTGG |  |  |
