# Supplementary material for: Prevalence of Ineffective Haplotypes at the Rice Blast Resistance (R) Gene Loci in Chinese Elite Hybrid Rice Varieties Revealed by Sequence-Based Molecular Diagnosis
Source: Rice (N Y). 2020 Jan 30;13:6. doi: 10.1186/s12284-020-0367-x (PMC6990218; doi:10.1186/s12284-020-0367-x)
Supplement: Supplementary file 4 — Additional file 4: Figure S1. Validation of R-gene specific primers for PCR amplification. [file 12284_2020_367_MOESM4_ESM.pptx]

## Slide 1
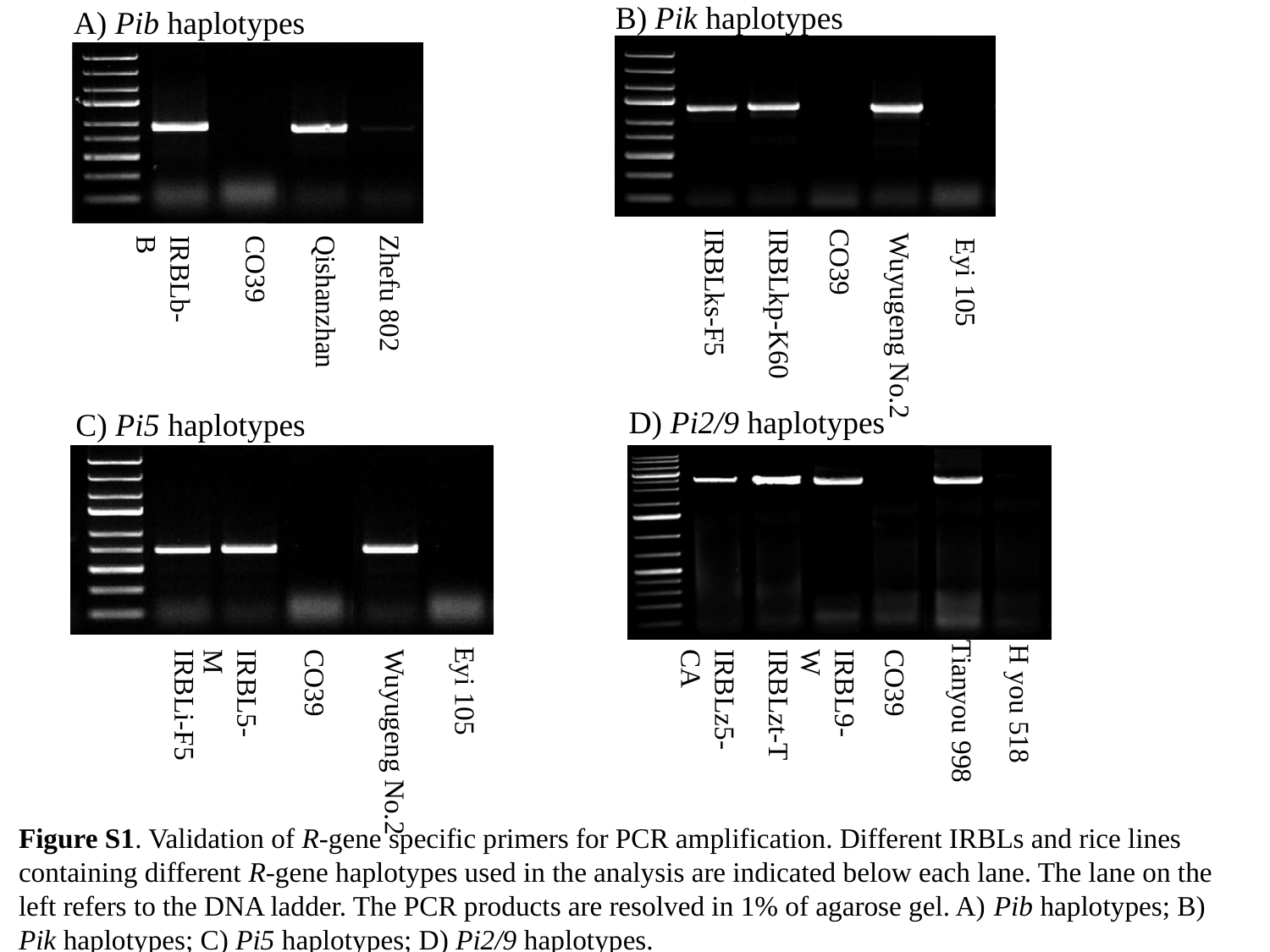

B) Pik haplotypes
A) Pib haplotypes
IRBLb-B
CO39
Qishanzhan
Zhefu 802
IRBLkp-K60
IRBLks-F5
CO39
Wuyugeng No.2
Eyi 105
D) Pi2/9 haplotypes
C) Pi5 haplotypes
Eyi 105
IRBLi-F5
IRBL5-M
CO39
Wuyugeng No.2
IRBLz5-CA
IRBLzt-T
IRBL9-W
CO39
H you 518
Tianyou 998
Figure S1. Validation of R-gene specific primers for PCR amplification. Different IRBLs and rice lines containing different R-gene haplotypes used in the analysis are indicated below each lane. The lane on the left refers to the DNA ladder. The PCR products are resolved in 1% of agarose gel. A) Pib haplotypes; B) Pik haplotypes; C) Pi5 haplotypes; D) Pi2/9 haplotypes.
