# Supplementary material for: Prevalence of Ineffective Haplotypes at the Rice Blast Resistance (R) Gene Loci in Chinese Elite Hybrid Rice Varieties Revealed by Sequence-Based Molecular Diagnosis
Source: Rice (N Y). 2020 Jan 30;13:6. doi: 10.1186/s12284-020-0367-x (PMC6990218; doi:10.1186/s12284-020-0367-x)
Supplement: Supplementary file 5 — Additional file 5: Figure S2. Disproportional distribution of different known R genes in rice varieties. [file 12284_2020_367_MOESM5_ESM.pptx]

## Slide 1
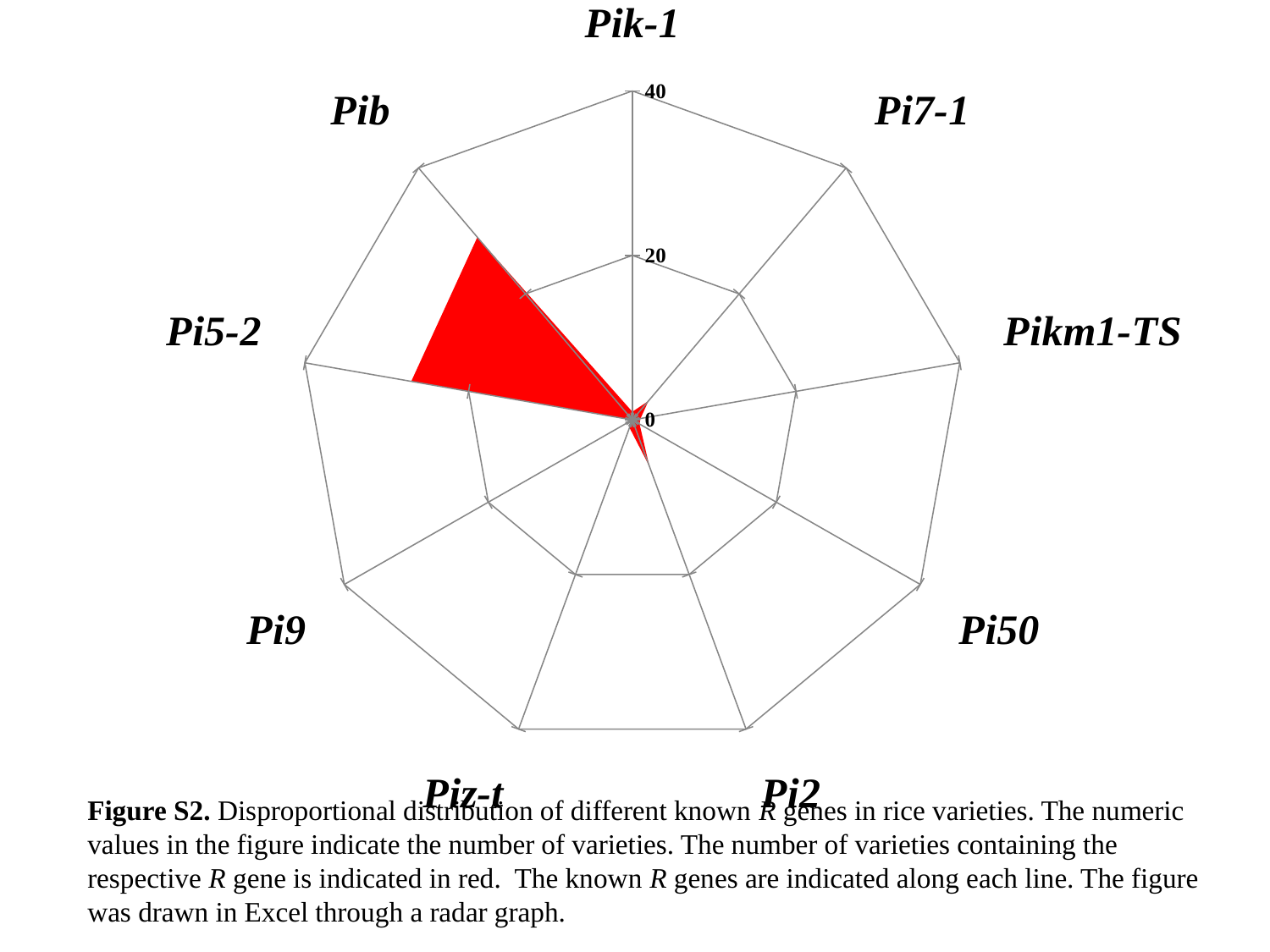

### Chart
| Category | |
|---|---|
| Pik-1 | 1.0 |
| Pi7-1 | 3.0 |
| Pikm1-TS | 1.0 |
| Pi50 | 1.0 |
| Pi2 | 6.0 |
| Piz-t | 1.0 |
| Pi9 | 0.0 |
| Pi5-2 | 27.0 |
| Pib | 29.0 |Figure S2. Disproportional distribution of different known R genes in rice varieties. The numeric values in the figure indicate the number of varieties. The number of varieties containing the respective R gene is indicated in red. The known R genes are indicated along each line. The figure was drawn in Excel through a radar graph.
