# Supplementary material for: Prevalence of Ineffective Haplotypes at the Rice Blast Resistance (R) Gene Loci in Chinese Elite Hybrid Rice Varieties Revealed by Sequence-Based Molecular Diagnosis
Source: Rice (N Y). 2020 Jan 30;13:6. doi: 10.1186/s12284-020-0367-x (PMC6990218; doi:10.1186/s12284-020-0367-x)
Supplement: Supplementary file 7 — Additional file 7: Figure S3. Resistance frequency of varieties in 1994 (A) and 2015 (B). [file 12284_2020_367_MOESM7_ESM.pptx]

## Slide 1
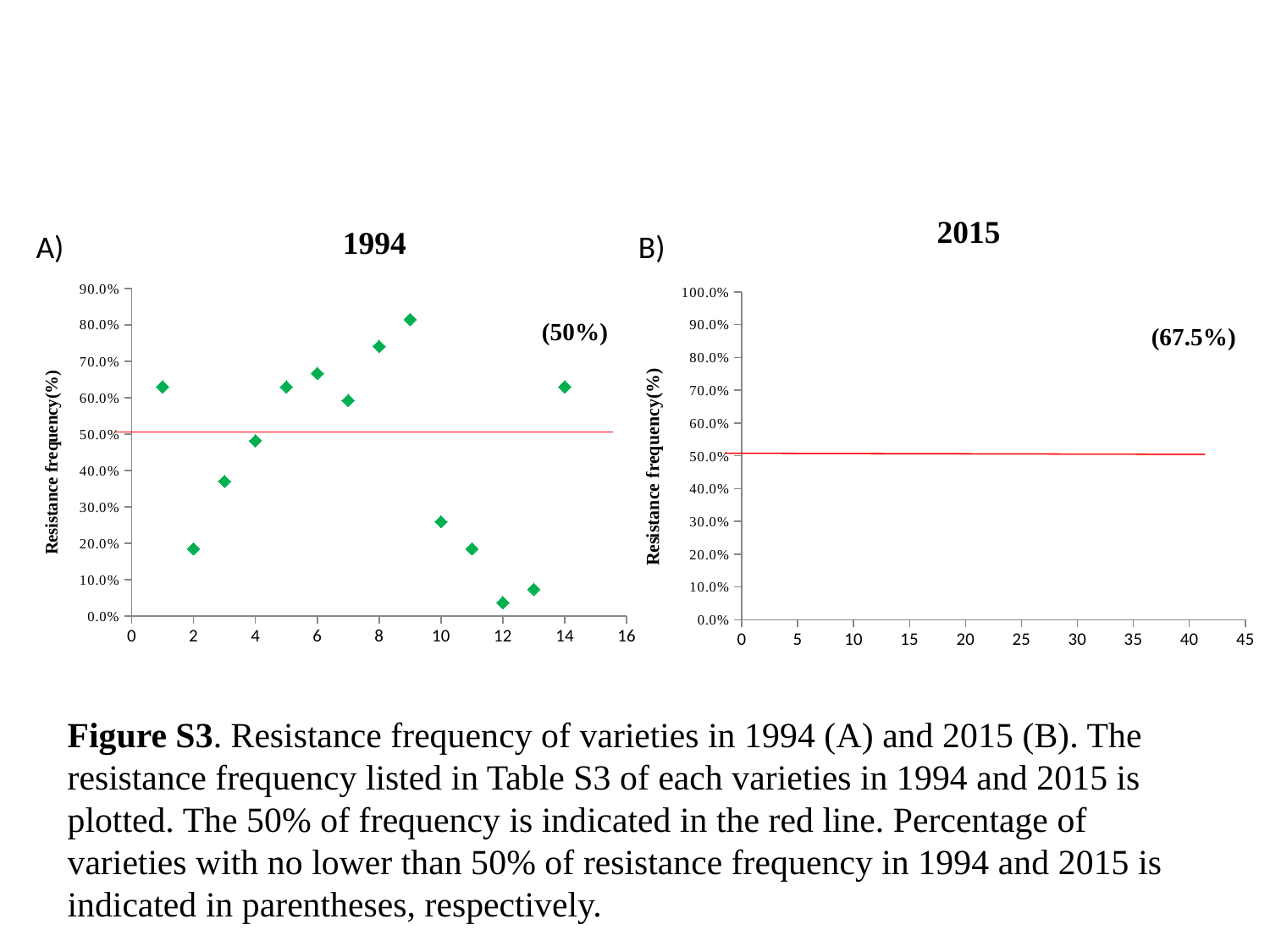

2015
1994
A)
B)
### Chart
| Category | |
|---|---|
### Chart
| Category | |
|---|---|(50%)
(67.5%)
Figure S3. Resistance frequency of varieties in 1994 (A) and 2015 (B). The resistance frequency listed in Table S3 of each varieties in 1994 and 2015 is plotted. The 50% of frequency is indicated in the red line. Percentage of varieties with no lower than 50% of resistance frequency in 1994 and 2015 is indicated in parentheses, respectively.
