# Supplementary material for: Prevalence of Ineffective Haplotypes at the Rice Blast Resistance (R) Gene Loci in Chinese Elite Hybrid Rice Varieties Revealed by Sequence-Based Molecular Diagnosis
Source: Rice (N Y). 2020 Jan 30;13:6. doi: 10.1186/s12284-020-0367-x (PMC6990218; doi:10.1186/s12284-020-0367-x)
Supplement: Supplementary file 8 — Additional file 8: Figure S4. Deduction of contribution of known and unknown R genes to the rice varieties. [file 12284_2020_367_MOESM8_ESM.pptx]

## Slide 1
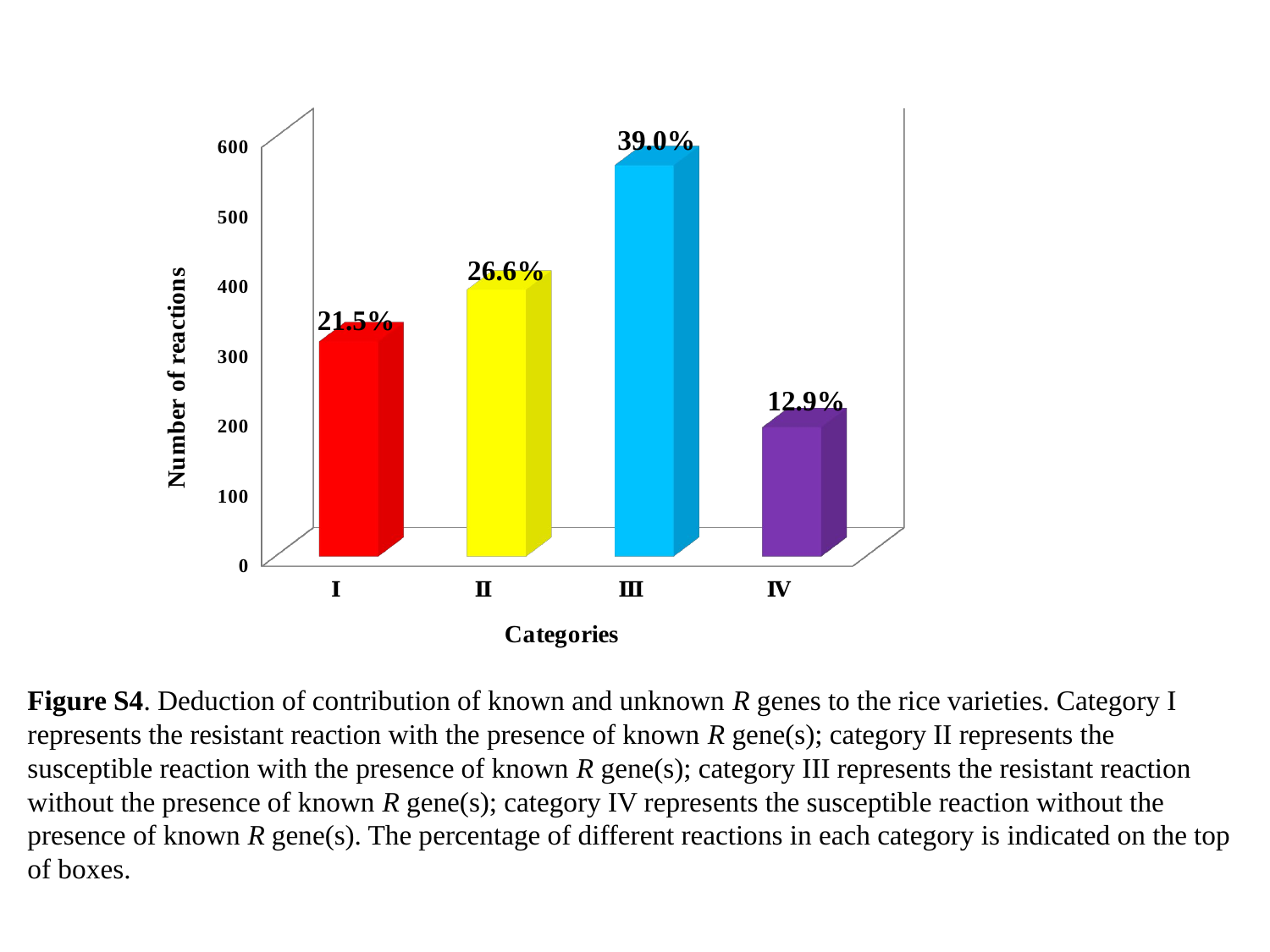

[unsupported chart]
39.0%
26.6%
21.5%
12.9%
Figure S4. Deduction of contribution of known and unknown R genes to the rice varieties. Category I represents the resistant reaction with the presence of known R gene(s); category II represents the susceptible reaction with the presence of known R gene(s); category III represents the resistant reaction without the presence of known R gene(s); category IV represents the susceptible reaction without the presence of known R gene(s). The percentage of different reactions in each category is indicated on the top of boxes.
